# Persistent Strongyloidiasis Complicated by Recurrent Meningitis in an HTLV Seropositive Peruvian Migrant Resettled in Italy

**DOI:** 10.4269/ajtmh.14-0716

**Published:** 2015-06-03

**Authors:** Lorenzo Zammarchi, Francesca Montagnani, Giacinta Tordini, Eduardo Gotuzzo, Zeno Bisoffi, Alessandro Bartoloni, Andrea De Luca

**Affiliations:** Clinica Malattie Infettive, Dipartimento di Medicina Sperimentale e Clinica, Università Degli Studi di Firenze, Largo Brambilla 3, 50134 Firenze, Italy; UOC Malattie Infettive Universitarie, UOC Microbiologia e Virologia, Dipartimento di Medicina Interna e Specialistica, Azienda Ospedaliera Universitaria Senese, Siena, Italy; Dipartimento di Biotecnologie Mediche, Università di Siena, Siena, Italy; Instituto de Medicina Tropical “Alexander von Humboldt,” Universidad Peruana Cayetano Heredia, Lima, Peru; Ospedale Sacro Cuore, Centro per le Malattie Tropicali, Negrar, Verona, Italy

## Abstract

We describe a case of persistent strongyloidiasis complicated by recurrent meningitis, in a human T cell lymphotropic virus type 1 (HTLV-1) seropositive Peruvian migrant adult resettled in Italy. He was admitted with signs and symptoms of acute bacterial meningitis, reporting four other meningitis episodes in the past 6 years, with an etiological diagnosis of *Escherichia coli* and *Enterococcus faecium* in two cases. He had been previously treated with several antihelmintic regimens not including ivermectin, without eradication of strongyloidiasis, and he had never been tested for HTLV before. During the described episode, the patient was treated for meningitis with broad-spectrum antibiotic therapy and 200 μg/kg/dose oral ivermectin once daily on day 1, 2, 15 and 16 with full recovery and no further episodes of meningitis. The presented case underlines several critical points concerning the management of poorly known neglected diseases such as strongyloidiasis and HTLV infection in low-endemic areas. Despite several admissions for meningitis and strongyloidiasis, the parasitic infection was not adequately treated and the patient was not previously tested for HTLV. The supply of ivermectin and the choice of treatment scheme was challenging since ivermectin is not approved in Italy and there are no standardized guidelines for the treatment of severe strongyloidiasis in HTLV seropositive subjects.

## Introduction

Strongyloidiasis is a nematode infection caused by *Strongyloides stercoralis*, widely distributed in tropical and subtropical areas, with some foci of low endemicity even in temperate areas.^1^ The infection is acquired primarily by walking bare foot on ground contaminated with infective larvae that are eliminated with stools by parasitized subjects. Once the subject has been infected, in the absence of appropriate treatment, *S. stercoralis* is able to persist indefinitely in the host through a so called autoinfective cycle. The infection is frequently unrecognized because it can be completely asymptomatic; or it may cause mild and nonspecific clinical complains such as itching, gastrointestinal discomfort, respiratory symptoms; or it can be responsible of asymptomatic eosinophilia.^1^ Moreover the diagnosis is difficult because it requires a combination of dedicated techniques such as blood agar plate culture of stool and specific serology since the direct parasitological examination of stool is insensitive.^2^ Even more challenging is the diagnosis in immunocompromised patients because serological tests may be negative.^2^

A peculiar characteristic of *S. stercoralis* is its ability to act as an opportunistic agent in immunocompromised subjects in whom it can cause hyperinfection syndrome and disseminated strongyloidiasis, two conditions with a high fatality rate.^3^

Hyperinfection syndrome is characterized by the presence of larvae confined to lungs and gastrointestinal tract, but with signs/symptoms of severe diseases in relation to elevated number of larvae, whereas disseminated strongyloidiasis is defined in presence of larvae in any organ, other than the respiratory and the gastrointestinal tracts.^3^

In such circumstances, the massive passage of intestinal bacteria through the bowel wall can be responsible for severe invasive bacterial diseases such as meningitis, which is the most frequent central nervous system manifestation of disseminated strongyloidiasis.^4^

Human T cell lymphotropic virus type 1 (HTLV-1) is a retrovirus highly prevalent in some areas of Japan, Papua New Guinea, South America, and West Africa.^5^ It is transmitted through mother-to-child route (mainly through breast-feeding), through blood transfusions, and through sexual route causing a chronic infection. Only about 10% HTLV-1-infected subjects develop a disorder related to the virus during their life, including adult T-cell leukemia/lymphoma, HTLV-1-associated myelopathy, uveitis, and opportunistic infections (including strongyloidiasis). As a matter of fact, HTLV-1 acts as immunosuppressant reducing the ability of the infected host to mount an adequate immune response to organisms that require a Th2-dependent response.^5^,^6^

## Case report

On March 14, 2014, a 35-year-old Peruvian man, body mass index (BMI) = 24.9, was admitted to the University Division of Infectious Diseases at Siena University Hospital (Siena, Italy) because of acute onset of severe headache, mental confusion, vomiting, and abdominal pain.

The patient had migrated to Italy from Peru 7 years before and he had never traveled back to his country of origin. Since 2008, he was admitted four times to different Italian hospitals with meningitis. In the first two instances, cerebrospinal fluid (CSF) culture yielded *Escherichia coli* and *Enterococcus faecium*, respectively, while two other episodes were diagnosed clinically since repeated attempts to perform lumbar puncture failed. During the first episode of meningitis, because of concomitant severe abdominal pain, he underwent an explorative laparotomy that did not reveal any abnormal finding. *S. stercoralis* larvae were found in stool microscopy during the first three episodes of meningitis. He was treated with different antihelmintic regimens, namely mebendazole 500 mg qid for 10 days, thiabendazole 500 mg bid for 5 days, albendazole 200 mg bid for 6 days and another course of albendazole with unspecified dosage and duration.

Physical examination revealed fever (body temperature 38°C), neck stiffness, positive bilateral Lasègue sign, and red dermographism. Blood tests were normal except for 18,060 leukocytes/μL (80% neutrophils) and C-reactive protein 1.01 mg/dL (reference value < 0.5 mg/dL).

Chest x-ray, abdominal ultrasound, and brain computed tomography (CT) scan did not reveal any alteration. Several attempt to lumbar puncture failed, because of difficulties related to anatomical conformation.

Parasitological stool examinations showed *S. stercoralis* larvae both in microscopic examination of three stool samples collected on alternate days and in Koga-agar plate culture. In brief, three stool samples were collected on alternate days in the concentration system of intestinal parasites, Paraprep S Gold Plus Kit (DiaMondial, Sées, France), and processed according to the manufacturer's instructions. Microscopic examination of the sediment revealed the presence of numerous *S. stercoralis* rhabditoid larvae in all the three samples. A modified method for *S. stercoralis* agar plate culture was performed as reported: a stool sample was cultured on multiple Mueller Hinton 2 agar plates (Biomerieux, Durham, NC); plates were sealed, incubated for 2–7 days at 22°C, protected from light. Plates were daily checked for tracks left by larvae and suspicious areas observed under a stereomicroscope (dissection microscope). After the first 18 hours incubation, *S. stercoralis* rhabditoid larvae were observed on the edge of the inoculum.

Although serology (SeroELISA *Strongyloides* IgG; IVD Research Carlsbad, CA)^7^ was negative.

HTLV antibodies were tested by a commercial chemiluminescent microparticle immunoassay (Architect rHTLV-I/II assay; Abbott, Wiesbaden-Delkenheim, Germany), according to manufacturer's instructions, and resulted strongly positive (index 128.20, cutoff value for positivity = 1). No other cause of acquired or congenital immunodeficiency was revealed. HIV ELISA test was negative, serum immunoglobulins were within normal range, and so were complement dosage and lymphocytic subset count.

*Strongyloides* serology was performed again at the Center for Tropical Diseases, Sacro Cuore Hospital, Negrar, Verona, Italy, with an in-house indirect immunofluorescence antibody test (IFAT)^8^ (negative), and with a commercial ELISA (Bordier ELISA; Bordier Affinity Products, Switzerland)^9^ that showed a slight positivity (index 1.1, cutoff value for positivity = 1).

Blood cultures for bacteria and fungi performed on admission were negative. The patient was also screened for hepatitis B and C, *Toxocara canis*, *Trypanosoma cruzi*, echinococcus, and cysticercus serologies, which were all negative.

On admission, blood cultures were performed and empirical, broad-spectrum antimicrobial treatment was started (meropenem 2 g tid, vancomycin 1 g bid, and acyclovir 750 mg tid). Acyclovir was discontinued after 7 days as herpetic encephalitis was deemed very unlikely, vancomycin was stopped on 14th day, while meropenem was continued for a total of 3 weeks.

Strongyloidiasis could not be treated as the drug of choice, ivermectin, was not registered in Italy, and was requested from the local pharmacy but not retrieved in time before discharge.

On the 21st day, the patient was discharged in excellent clinical conditions. After discharge, patient was referred to the Tuscany Reference Center for Tropical Diseases, Azienda Ospedaliero-Universitaria Careggi, Florence (Italy), where a small stock of ivermectin was available.

The case was discussed within the “World Health Organization Strongyloidiasis Information Sharing Platform” (http://ezcollab.who.int/ntd/strongyloidiasis), which is an internet-based system accessed by invitation only, intended to share information and exchange opinions among the subcommunity members on key issues of strongyloidiasis, to decide the management strategy. Three different strategies were suggested by three different experts (Table 1).

After discussion, the patient was treated with a first cycle of 200 μg/kg/dose ivermectin once daily on day 1, 2, 15, and 16. The first dose was administered under medical surveillance, while the rest of the doses were delivered to the patient who completed the regimen at home. Follow-up with full blood cell count, *Strongyloides* serology, direct parasitological examination of stool, stool culture, and polymerase chain reaction (PCR) for *Strongyloides* were scheduled but, unfortunately, the patient did not present to any follow-up appointment despite several telephone reminders and attempts to reschedule the follow-up visits. After 6 months of ivermectin treatment, the patient did not complain of any symptom at telephone follow-up. A parasitological screening of all family members (the wife and two children) was also repeatedly suggested, without any success so far.

## Discussion

The case presented highlights the possibility of recurrent meningitis due to intestinal bacteria in HTLV-1-infected patients with strongyloidiasis refractory to treatment.

Even though a test to distinguish among HLTV-1 and HTLV-2, such as a Western blot, was not performed, the patient was most likely infected with HTLV-1 given the epidemiological distribution of the virus in the patient's country of origin^10^ and because, unlike HTLV-2, HTLV-1 only has been associated with complicated strongyloidiasis.^6^,^11^ *Strongyloides* hyperinfection is strongly associated with HTLV-1 infection in Peru.^12^

Meningitis in association with strongyloidiasis is usually due to intestinal pathogens and diagnosed in severely immunocompromised patients who develop disseminated strongyloidiasis,^4^ a condition with a fatality rate of about 70%.^3^

Recurrent episodes of bacterial meningitis in patients with strongyloidiasis are reported in subjects presenting predisposing factors, such as organ transplant,^13^ corticosteroid use,^14^ diabetes,^14^ and HTLV-1 infection.^15^

The association of strongyloidiasis and meningitis is not infrequent in some endemic areas where *S. stercoralis* and HTLV-1 are co-endemic, such as subtropical areas of Japan. In a recent case series, 33 meningitis episodes in 21 patients with strongyloidiasis in a 10-year period were described, with five patients presenting recurrent episodes. Out of 21 patients, 16 were HTLV-1 positive, and all 5 patients with recurrent episodes were also HTLV-1 positive.^15^ In 19 out of 33 episodes described in this series, blood and/or CSF culture yielded intestinal bacteria (with *Klebsiella pneumoniae*, *Streptococcus bovis*, and *Escherichia coli* being the most frequently isolated), while 14 out of 33 cases were culture negative suggesting that the parasite itself may invade the meninges without any concurrent bacteria, and that *S. stercoralis* alone may induce the intense inflammatory changes that mimic the CSF findings of bacterial meningitis, as already hypothesized by Kishaba and others.^16^

HTLV-1 interferes with Th2 cell responses to *S. stercoralis*, including eosinophils activation and production of interleukins and IgE.^17^ Moreover, a poor response to antihelmintic treatment (ivermectin, albendazole, and thiabendazole) of *S. stercoralis* has been observed in HTLV-1-infected individuals.^6^

In uncomplicated strongyloidiasis, ivermectin is the drug of choice because it is more effective than albendazole^18^ and better tolerated than thiabendazole,^19^ although the optimal dosage and schedule of ivermectin has yet to be determined.

Management strategies for severe case of strongyloidiasis, such as the one reported in this article, are not yet standardized. A recent systematic review of literature based on case reports and short case series of severe strongyloidiasis outline a “Babylon of different treatment schemes.”^3^ Even the different suggestions for our case provided by the consulted experts through the web-based platform confirm the lack of a standardized treatment approach to this condition. However, all consulted experts agreed on including repeated doses of ivermectin in the treatment strategy and on the need for a strict follow-up to assess cure. As far as follow-up is concerned, a combination of all available laboratory techniques including stool culture, serology, and PCR is probably the best strategy to achieve the highest sensitivity to detect a treatment failure, even if the interpretation of serology may be critical and evidence for the usefulness of PCR in this context is lacking.^20^ Our case underlines several crucial points concerning the management of poorly known neglected infectious diseases such as strongyloidiasis and HTLV-1 in a low-endemic area in the “global health era.”

The migrant patient was repeatedly admitted to different Italian hospitals for meningitis and strongyloidiasis, but the two conditions had never been causally linked. He had never been tested for HTLV-1 nor treated with ivermectin, the drug of choice for strongyloidiasis. He underwent an invasive, explorative laparotomy because of severe abdominal pain most likely due to strongyloidiasis, which could have been avoided if the parasitic infection had been properly suspected and managed.

Our case also confirms the possibility of false-negative *Strongyloides* serology in immunocompromised patients^21^–^23^ with severe forms of strongyloidiasis demonstrating the irreplaceable and complementary role of appropriate parasitological investigation (particularly stool culture).

Our experience also underscores the difficulties in obtaining ivermectin that is not registered in some countries and that may be urgently needed to treat a severe infection.

Finally, we wish to underline the usefulness of the “World Health Organization Strongyloidiasis Information Sharing Platform,” the internet-based system that allowed to easily share opinions with experts on the management strategy.

On the basis of our case report and on the literature available, we suggest conclusive few, key take-home messages, as summarized in Table 2.

## Figures and Tables

**Table 1:** Management strategies for the patient described in the case report proposed by some of the members of the “World Health Organization Strongyloidiasis Information Sharing Platform” (http://ezcollab.who.int/ntd/strongyloidiasis)

Expert number	Proposed treatment strategy	Proposed follow-up strategy
Expert 1	Albendazole 400 mg bid plus ivermectin 200 μg/kg/dose once daily for two consecutive days a week for at least 1 month	Stool culture, PCR, serology
Expert 2	Ivermectin 200 μg/kg/dose once daily on day 1, 2, 15 and 16	Stool culture only
Expert 3	Ivermectin 200 μg/kg/dose once daily for 2–5 days (depending on the larval burden), repeat at days 14 and 15	Stool culture, PCR, serology
		Secondary prophylaxis may be required for some patients who fail therapy

PCR = polymerase chain reaction.

**Table 2:** Take-home message learned from this case

1) Clinicians working in non-endemic areas should be aware of neglected infectious diseases such as strongyloidiasis and HTLV-1, which, if associated, may determine a fatal outcome
2) Patients with meningitis due to intestinal bacteria should undergo serological and parasitological test for strongyloidiasis
3) Patients with meningitis and strongyloidiasis, as well as those with strongyloidiasis who fail to respond to antiparasitic treatment, should be tested for HTLV-1
4) In immunocompromised patients with strongyloidiasis, serology for *Strongyloides* may be falsely negative, and fecal-based tests, including culture for *Strongyloides*, are mandatory
5) Ivermectin should be made universally available for the treatment of strongyloidiasis
6) In HTLV-1 infected patients, efficacy of standard antihelmintic regimens is reduced, therefore strongyloidiasis must be treated aggressively

HTLV-1 = human T cell lymphotropic virus type 1.
